# Factor structure of The Opening Minds Stigma Scale for Health Care Providers and psychometric properties of its Hungarian version

**DOI:** 10.1186/s12888-020-02902-8

**Published:** 2020-10-12

**Authors:** Dorottya Őri, Sándor Rózsa, Péter Szocsics, Lajos Simon, György Purebl, Zsuzsa Győrffy

**Affiliations:** 1 Vadaskert Child and Adolescent Psychiatric Hospital, Budapest, Hungary; 2 Department of Psychiatry, Washington University School of Medicine, St. Louis, USA; 3 Institute of Experimental Medicine, Budapest, Hungary; 4 Department of Psychiatry and Psychotherapy, Semmelweis University, Budapest, Hungary; 5 Institute of Behavioural Sciences, Semmelweis University, Nagyvárad tér 4, Budapest, H-1089 Hungary

**Keywords:** Stigma, Scales, Measurement, Mental health related stigma, Attitudes, Psychometrics, Reliability, Psychiatrists

## Abstract

**Background:**

The Opening Minds Stigma Scale for Health Care Providers (OMS-HC) is a widely used questionnaire to measure the stigmatising attitudes of healthcare providers towards patients with mental health problems. The psychometric properties of the scale; however, have never been investigated in Hungary. We aimed to thoroughly explore the factor structure of the OMS-HC and examine the key psychometric properties of the Hungarian version.

**Methods:**

The OMS-HC is a self-report questionnaire that measures the overall stigmatising attitude by a total score, and three subscales can be calculated: Attitude, Disclosure and Help-seeking, and Social Distance. Our study population included specialists and trainees in adult and child psychiatry (*n* = 211). Exploratory and confirmatory factor analyses were performed, and higher-order factors were tested. We calculated the test-retest reliability on a subgroup of our sample (*n* = 31) with a follow-up period of 1 month. The concurrent validity of the scale was measured with the Mental Illness: Clinician’s Attitudes-4 scale (MICA-4).

**Results:**

Three factors were extracted based on a parallel-analysis. A bifactor solution (a general factor and three specific factors) showed an excellent model-fit (root mean square error of approximation = 0.025, comparative fit index = 0.961, and Tucker-Lewis index = 0.944). The model-based reliability was low; however, the general factor showed acceptable reliability (coefficient omega hierarchical = 0.56). The scale demonstrated a good concurrent validity with the MICA-4 [intraclass correlation coefficient (ICC) = 0.77]. The test-retest reliability was excellent for the general factor (ICC = 0.95) and good for the specific factors (ICC = 0.90, 0.88, and 0.84, respectively).

**Conclusions:**

The three dimensions of the OMS-HC was confirmed, and the scale was found to be an adequate measure of the stigmatising attitude in Hungary. The bifactor model is more favourable as compared to the three correlated factor model; however, despite the excellent internal *structure,* its model-based reliability was low.

## Background

Stigma is defined as a social rejection by presumed negative characteristics that leads to a “spoiled identity” according to Goffmann [1]. People with mental illness often face stigma in their everyday life [2] and also, unfortunately, when seeking medical help [3, 4]. Stigma causes negative consequences in their quality of life in terms of interpersonal relationships and institutional opportunities [5]. Several studies have shown that mental health professionals could express stigmatising attitudes towards their patients with mental illness [6–11]; however, to a lesser extent as compared with the other members of the society [12, 13]. Moreover, a significant reduction has been observed in the stigmatising attitude following anti-stigma interventions [14–16]. Kassam compared the stigmatising attitude of psychiatrists to that of other medical specialists in Canada, and the results revealed that the scores for psychiatrists on every measure were significantly lower than the scores for family physicians, rural and emergency rural physicians, anaesthetists, and surgeons [17]. The results of a Belgian study pointed out that the associative stigma of mental health care providers is related to more self-stigma and dissatisfaction of the clients besides the increased symptoms of burnout and less job satisfaction of mental health professionals themselves [18]. Thus, it is important to draw attention to the potentially deleterious impact of the stigmatising attitude of healthcare providers on their patients.

A cross-sectional international study that included a Hungarian sample as well on people with schizophrenia demonstrated that people with mental disorders experience disrespect and discrimination from healthcare providers [4]. The results have shown that patients from post-communist countries like Hungary felt more disrespected compared to other countries. However, no studies have examined the degree of stigma from the side of healthcare professionals towards their patients in Hungary. There is also a great need for a suitable questionnaire in Hungarian language for measuring the stigmatising attitudes and behaviours because it might serve as the first step of national anti-stigma interventions.

The Opening Minds Stigma Scale for Health Care Providers (OMS-HC) was developed in Canada within the framework of the “Opening Minds” anti-stigma initiative. It is a widely used self-report questionnaire that assesses various dimensions of the stigmatising attitude of healthcare providers towards their patients with mental health problems [17]. The initial version of the scale consisted of 20 items, and its factor structure showed a 2-factor solution with 12-items that appeared to be lacking the important social distance dimension of the stigma construct; therefore, the research group decided to investigate their scale further. Two years later, the factor structure of the scale was examined on a more representative population of healthcare providers with excessive involvement of physicians and nurses. According to their results, the 15-item version of the scale with three fixed factors (i.e. Attitude, Disclosure and Help-seeking, and Social Distance) exhibited a more stable solution. This version displayed a good construct validity and satisfactory internal consistencies (α = 0.67 to 0.79) [19]. Later-on, the psychometric properties of the scale has been tested in international studies, and it is a reliable and valid scale in Singapore [20], Italy [21], and Chile [22] on various populations of healthcare providers. The Singaporean research group applied a slight modification on the factor structure and proposed a 14-item version by excluding one item of the attitude subscale due to its poor factor loading. The results summary of the factor analyses on the 15-item OMS-HC in international studies is demonstrated in Table 1.

**Table 1:** Overview of the results of the factor analyses on the 15-item OMS-HC in international studies

Research group	Investigated population	Method	Results		Country
			Structure	Model fit indices	
Modgill et al., 2014 [19]	healthcare and social workers and medical students *n* = 1305	PCA	- 3 dimensional structure	–	Canada
Destrebecq et al., 2018 [21]	healthcare students *n* = 561	EFA	- 3 dimensional structure - Item 20 has poor factor loading on the Attitude factor	–	Italy
Chang et al., 2017 [20]	nurse and medical students *n* = 1002	ESEM	- 3 dimensional structure - Item 1 was deleted - Items 6, 7, 17 showed strong cross-loadings - Items 7, 17 loaded on different factors	RMSEA = 0.069 CFI = 0.948 TLI = 0.909	Singapore
Sapag et al., 2019 [22]	primary healthcare workers *n* = 803	SEM	- 3 dimensional structure	RMSEA = 0.052 CFI = 0.832 TLI = 0.798	Chile

*PCA* Principal component analysis, *EFA* Exploratory Factor analysis, *ESEM* Exploratory structural equation modelling (integration of EFA, confirmatory factor analysis and SEM), *SEM* Structural equation modelling, *RMSEA* Root mean square error of approximation, *CFI* Comparative fit index, *TLI* Tucker-Lewis Index.

## Methods

### Aims

Interestingly, the psychometric properties of the OMS-HC have been investigated on several different samples; however, only the original version of the scale has been studied on a sample that included practising psychiatrists. We aimed to examine the key psychometric properties of the Hungarian version of the 15-item OMS-HC on a population of trainees and specialists in child and adolescent and adult psychiatry.

### Study overview

This was a prospective, cross-sectional, observational study that applied an anonymous online survey designed to measure the stigmatising attitude of trainees and specialists in adult, as well as in child and adolescent psychiatry. Our research group contacted via e-mail and telephone a total of 50 adult and 10 child psychiatric inpatient services together with 52 adult and 17 child outpatient services across Hungary, serving both urban and rural areas. We directly sent the link of the survey to the head of the unit and requested to forward it to their psychiatrist colleagues. To maximise the reachable study population, we also shared the link of the survey through social media platforms and included it to the newsletter of the Hungarian Association of Psychiatric Trainees and the Hungarian Psychiatric Association.

### Participants

A total of *n* = 238 professionals in psychiatry responded to our survey. We analysed the data of *n* = 211 participants who completed the entire survey. Following the first round of the data collection, we disseminated the OMS-HC twice among the members of the Hungarian Association of Psychiatric Trainees with a 1 month difference between the two test administrations to be able to measure the intraclass correlation in order to assess the test-retest reliability. We used unique identifiers to maintain their anonymity and to be able to perform the repeated measures. Altogether *n* = 31 participants filled in the OMS-HC twice; therefore, we used the data of these people in the test-retest reliability measures.

### Measurements

#### Demographic data

The following information was gathered by using direct questions: age range, sex, years of experience in psychiatry, type and place of the working institute.

#### Stigmatising attitude

##### OMS-HC

The 15-item version of the Opening Minds Stigma Scale for Healthcare Providers is a self-report questionnaire that contains 15 statements describing feelings and opinions about people with mental health problems. This scale is different from the original 20-item version of the scale because items 2, 5, 11, 15, and 16 are not listed; however, as applied in the international studies, the order and the number of the items remained the same. In the survey, the subjects indicate on a 5-point Likert scale (1 = “strongly disagree” to 5 = “strongly agree”) the extent they identify themselves with the given statement. Five items (item 3, 8, 9, 10, and 19) are reverse coded (1 = “strongly agree” to 5 = “strongly disagree”). The overall stigmatising attitude of the participants is described with the total score of the scale (minimum of 15, and a maximum of 75 points). Besides the total score, three dimensions can be calculated by evaluating the three subscales of the questionnaire (Attitude, Disclosure and Help-seeking, and Social Distance). Higher scores on a subscale and higher total scores reflect a more stigmatising attitude.

The English version of the OMS-HC (Supplementary file 1) was first translated into Hungarian, and then back-translated to English by a qualified specialist in English Medical and Health Sciences. An iterative procedure was used to resolve any discrepancies between the original and the back-translated versions of the scale, and then a focus group of 6 psychiatrists did the concept checking. The final Hungarian version (Supplementary file 2) was then sent to the participants.

##### MICA-4

The Mental Illness: Clinician’s Attitudes-4 (MICA-4) [23] scale was applied to measure the convergent validity of the OMS-HC. This scale contains 16 statements and continuously measures the attitude towards people with mental health problems. The total score ranges from 16 (least stigmatising) to 96 (most stigmatising). The MICA-4 scale was translated similarly to the translation process of the OMS-HC; a British-Hungarian bilingual researcher helped our group in the back-translation process in line with the translation guideline of the Indigo Network. (http://www.indigo-group.org).

### Statistical approach

The demographic data are expressed as sample size (n) with a percentage (%). Since our data did not follow the normal distribution we performed non-parametric tests. For comparison of two groups we used the Mann-Whitney U test; whereas for comparison of more than two groups we applied one-way ANOVA test. We defined the number of factors to be extracted via parallel analysis that compares the progressive eigenvalues from the given data matrix to that of a simulated data matrix by using random data of the same size [24]. Polychoric correlations were used because the data did not follow the normal distribution due to the ordinal nature of the responses. Prior to the exploratory factor analysis (EFA), Bartlett’s Test of Sphericity was applied to ensure the non-randomness of the correlation matrix (*p*-value should be < 0.05), while the Kaiser-Meyer-Olkin (KMO) measure of sampling adequacy was calculated to ensure that the matrices were suitable for the analysis (should be > 0.60) [25]. We employed the unweighted least squares method with geomin rotation, as well as the hierarchical Schmid-Leiman solution to identify the factor structure of the scale [26]. Confirmatory factor analysis (CFA) was done to examine the fit of the original and the proposed models. Since the variables were non-parametric, we performed the CFA by using a robust estimator (the maximum likelihood estimation with robust standard errors and a mean- and variance adjusted, MLMV) that appropriately corrects for the standard errors of the parameters. To evaluate the model fit, we calculated the following indices and adopted the generally recommended criteria: chi-square (χ^2^), degree of freedom (df), χ^2^/df (2.0–5.0) [25], root mean square error of approximation (RMSEA, < 0.06) [27], comparative fit index (CFI, > 0.95) [27], Tucker-Lewis Index (TLI, > 0.95) [27]. Model-based reliability was evaluated by using the coefficient omega hierarchical (ωH), the explained common variance (ECV), and the percent of uncontaminated correlations (PUC). These provide evidence that the total score and the subscale scores genuinely represent the target constructs of interest. Since there has been no accepted cut-off value for the evaluation of the ωH, we applied what Reise et al. suggested: it should be greater than 0.50, and ideally more than 0.75 [28]. In addition, when the PUC values are higher than 0.80, the general ECV values are less important in predicting bias. If the PUC values are lower than 0.80, the general ECV values greater than 0.60 and the ωH values greater than 0.70 suggest that the presence of the multidimensionality is not severe enough to disqualify the interpretation of the instrument as primarily unidimensional [28]. In order to compare the model-based reliability and the internal consistency measures, the Cronbach’s α coefficients were calculated for the correlated factor model (0.70–0.95) [29]. The intercorrelations among the specific factors and the general factor were assessed by using the Spearman’s correlation (Spearman’s r = 0.00–0.19 very weak, 0.20–0.39 weak, 0.40–0.59 moderate, 0.60–0.79 strong 0.80–1.00 very strong). In order to describe the test-retest reliability, the intraclass correlation coefficient (ICC) was calculated along with the 95% confidence intervals based on a mean-rating (k = 2), absolute-agreement, two-way mixed-effects model (ICC < 0.50 poor, 0.50–0.75 moderate, 0.75–0.90 good, > 0.90 excellent) [30]. For concurrent validity measures, we also used a two-way mixed effects model with absolute agreement. We calculated the ICC estimates and their 95% confidence intervals to test the agreement between the scale and the MICA-4. The following softwares were used for the statistical analyses: IBM SPSS 25 (Apache Software Foundation, USA), and MPlus 6.12 (Muthen and Muthen, USA).

## Results

A total of *n* = 238 professionals in psychiatry responded to our survey. The completion rate was 89%; thus, we analysed the data of *n* = 211 participants who completed the entire survey. The majority of the participants were female (*n* = 161, 76%) with the overrepresentation of young colleagues between 24 and 35 years of age (*n* = 114, 54%), and with 0–5 years of experience (*n* = 84, 40%). Most of them worked in adult psychiatry (*n* = 135, 64%), and as a specialist (*n* = 121, 57%). In approximately two-thirds of the cases, they worked at inpatient services (*n* = 139, 66%), and nearly two-thirds of the sample has a workplace in Budapest, which is the capital city of Hungary (*n* = 131, 62%).

There were no statistically significant differences between males and females in the total score [30 (28–35) vs 32 (28–35), *p* = 0.12, Cohen’s d = 0.25]. Similarly, the total score did not differ relevantly among participants with different years of experience (0–5 years, 5–10 years, 11–20 years, 21–30 years, 31–40 years, > 40 years): one-way ANOVA *p* value = 0.88 Cohen’s f = 0.22.

In the first step, we tested the original three-factor model of the 15-item scale by CFA (Table 2). The absolute fit indices (χ^2^ and RMSEA) were within the predefined ranges; however, the incremental fit indices (CFI and TLI) were lower than acceptable; therefore, we sought to explore the structure further.

**Table 2:** Results of the confirmatory factor analysis of the OMS-HC

	χ^**2**^	χ^**2**^/df	***RMSEA***	95% CI of RMSEA	CFI	TLI
Original 15-item scale	129.602	1.45	0.048	0.030–0.065	0.818	0.780
Unidimensional 15-item scale	173.562	1.93	0.066	0.051–0.081	0.642	0.583
3 correlated factors based on EFA results (15 items)	123.479	1.45	0.045	0.024–0.062	0.844	0.812
3 correlated factors with the deletion of 1 item based on EFA results (14 items)	103.475	1.39	0.043	0.021–0.062	0.867	0.836
Bifactor solution (14 items)	71.055	1.13	0.025	0.000–0.050	0.961	0.944

The confirmatory factor analysis was performed by using the maximum likelihood estimation with robust standard errors and a mean- and variance adjusted.

χ^2^: chi-square, df: the degree of freedom, RMSEA: root mean square error of approximation, *CI* Confidence interval, *CFI* Comparative fit index, *TLI* Tucker-Lewis Index.

In the next step, we performed an EFA; Bartlett’s Test of Sphericity (*p* < 0.0001) and the KMO measure of sampling adequacy (0.72) suggested that the data were suitable for it. To decide the number of factors to be extracted, we performed a parallel analysis by creating 500 randomly generated correlation matrices. The eigenvalues were 3.75, 1.64, and 1.56 that accounted for 46% of the total variance (25, 11, and 10%; respectively). Consequently, the first factor explains a relatively large proportion of the variance, indicating that all items might belong to a single dimension. However, the parallel analysis suggested the extraction of three factors because they account for a sufficient amount of variance.

To find the most appropriate model, we tested both the unidimensional and the 3-dimensional models. As presented in Table 3, if three correlated factors were extracted, the pattern matrix showed a relatively clear 3-factor solution, which is similar to that of the original 15-item model except for three items (14, 18 and 19). Items 18 and 19 exhibited cross-loadings on the Attitude and Social distance factors. Since item 14 expressed a poor loading on all of the three factors, we decided to remove it from the scale and the subsequent CFA analyses.

**Table 3:** The factor structure of the 15-item version of the OMS-HC

Items	Original subscale	Factors		
		1 (Disclosure)	2 (Social distance)	3 (Attitude)
4	Disclosure	**0.597**	0.040	0.127
6	Disclosure	**0.549**	0.233	0.211
7	Disclosure	**0.526**	0.072	0.245
10	Disclosure	**0.499**	0.275	0.065
3	Social distance	0.024	**0.474**	0.247
8	Social distance	0.097	**0.501**	0.183
9	Social distance	0.168	**0.694**	0.100
17	Social distance	0.273	**0.565**	0.048
18	Attitude	0.064	**0.319**	0.269
1	Attitude	0.081	0.051	**0.430**
12	Attitude	0.141	0.278	**0.411**
13	Attitude	0.095	0.029	**0.545**
19	Social distance	0.094	**0.322**	**0.447**
20	Attitude	0.192	0.054	**0.490**
14	Attitude	0.186	0.181	0.248

The unweighted least squares method was used with geomin rotation. Factor loadings higher than 0.3 are highlighted in bold.

To further investigate the model, higher-order factors were tested by using the Schmid-Leiman solution, which involves transforming an oblique factor solution into an orthogonal version of higher-order and primary factors. This bifactor solution models a general factor called “overall stigmatising attitude” along with the three sub-factors (i.e. the three subscales): Attitude, Disclosure and Help-seeking, and Social distance. In this bifactor model, each variable loads on two factors, one general and one specific.

The next step was to perform CFA to evaluate the model fit of the unidimensional, the three correlated factor, and the bifactor solutions. As presented in Table 2, the fit indices of the unidimensional model were not in the acceptable range, while the three-factor solutions displayed a borderline acceptable fit. The bifactor model; however, showed a good fit and found to be the most appropriate one.

In the final step, we tested the model-based reliability. Both the general, as well as the specific factors exhibited a poor model-based reliability: general factor (ECV = 0.43, ωH = 0.56), Attitude (ECV = 0.18, ωH = 0.37), Disclosure and Help-seeking (ECV = 0.19, ωH = 0.44), Social distance (ECV = 0.19, ωH = 0.37). The PUC value for the bifactor model with the three specific factors was 0.71.

To ensure the comparison of the reliability of the bifactor and the three correlated factor models, we also computed the Cronbach’s alpha coefficients: 0.73 (total score), 0.54 (Attitude), 0.63 (Disclosure and Help-seeking), and 0.66 (Social distance).

The correlations between the specific factors and the general factor of the OMS-HC were strong (Attitude: *r =* 0.68, *p* < 0.0001; Disclosure and Help-seeking: *r =* 0.69, *p* < 0.0001; Social distance: *r =* 0.73, *p* < 0.0001). The specific factors also correlated statistically significant with each other (Attitude and Disclosure and Help-seeking: *r =* 0.22, *p* = 0.002; Attitude and Social distance: *r =* 0.33, *p* < 0.0001; Disclosure and Help-seeking and Social distance: *r =* 0.24, *p* < 0.0001).

A subsample of the subjects (*n* = 31) completed the survey twice with a median follow-up period of 1 month (median 28 [26–30] days). Table 4 demonstrates that the test-retest reliability between the two administrations of the OMS-HC was excellent in case of the general factor and good in case of the specific factors.

**Table 4:** Test-retest reliability measures

	ICC	95% CI of ICC
**Attitude**	0.90	0.80–0.95
**Disclosure and** **Help-seeking**	0.88	0.76–0.94
**Social distance**	0.84	0.66–0.92
**Scale**	0.95	0.89–0.97

Test-retest reliability was measured by intraclass correlation coefficients and their 95% confidence intervals using an absolute-agreement, two-way mixed-effects model

*ICC* Intraclass correlation coefficient, *CI* Confidence interval

The comparative examination of the OMS-HC with the MICA-4 total score resulted in an ICC value of 0.77 [95% CI 0.11 to 0.92] that suggests a good relationship.

## Discussion

In this study, we examined the psychometric properties of the 15-item version of the OMS-HC on a sample of trainees and specialists in child as well as in adult psychiatry.

The construct of the OMS-HC has already been studied in some countries [17, 19–22]; however, this is the first study that examines its 15-item version on practising psychiatrists. Despite the widespread use of the OMS-HC, its latent factor structure has only been examined in three countries [19–21]. Moreover, only two research groups have performed CFA on the scale items (see Table 1), and none of them has investigated its structure further to higher-order solutions. The results of the present study partially support the theoretical approach that the OMS-HC consists of Attitude, Disclosure and Help-seeking, and Social distance subscales [19]; moreover, we set up new perspectives and gave insights into the interpretations of the OMS-HC scores and dimensions.

Since our confirmatory factor analysis on the 15-item version of the scale proposed by Modgill et al. [19] indicated a good fit based on the absolute fit indices but a poor fit based on the relative indices, we aimed to explore its factor structure further. Our results are in correspondence with the findings of the CFA in the Chilean study [22], which showed approximately the same model fit on the population of various mental health providers; however, they did not examine the latent factor structure. The parallel analysis suggested that three factors should be extracted and the results of the EFA also indicated the three dimensions to be the relevant constructs of the scale. These support the findings of the Canadian research group who reduced the original 20-item scale [17] to the 15-item version [19]. In addition, item 14 (*„More than half of people with mental illness don’t try hard enough to get better.”)* showed poor loadings across all of the three factors; therefore, we removed it from the scale. This item loaded on the attitude factor in the Canadian study [19]; however, it was not among the strongest items either due to its lower factor loading compared to other items. Item 14 differs from other items in terms of content because it gives information about the responsibility of people with mental health problems, which should not be directly linked to the attitude of health care providers. Similarly, the Singaporean research group performed an EFA as well. They found that item 1 had been expressing a poor factor loading; therefore, they also eliminated one item [20]. To further explore the structure, the higher-order factors were also tested. The CFA results exhibited an excellent model-fit in case of the bifactor solution. In this model, the items load on both the general factor and on one of the three specific factors. The general factor entitled as the “overall stigmatising attitude” accounts for all of the elements common to the specific factors. The three specific factors called “Attitude”, “Disclosure and Help-seeking”, and “Social distance” reflect the three factors in a correlated model. The fit indices of the bifactor model were more favourable as compared to those that of the three correlated factor model. While the internal *structure is excellent of the bifactor solution,* its model-based reliability was found to be poor. The general factor exhibited the minimum acceptable reliability [28]; however, the specific factors did not possess sufficient reliable variance for the interpretation. In contrast, the relative fit indices of the correlated trait model were not in the acceptable ranges; although, its internal consistency measures indicated a reliable total score and borderline acceptable subscale scores. It should be highlighted that the Cronbach’s alpha coefficients of the total and the subscale scores were similar to those that were described in Singapore [20] and Chile [22]. In Canada the coefficients were higher than in our study [19]. The internal consistency was found to be good in the Italian study [21].

Based on the above, a question might arise whether the correlated factor model with the three subscales and a total score or the bifactor model with a general factor and three orthogonal specific factors is the most appropriate approach. Actually, it depends on what would we like to measure with the scale. We highlight that both options have specific advantages and disadvantages. We recommend using the bifactor model if the factor structure is in the scope, and the exploration of the model is important. In this case, it is reasonable to measure the overall stigmatising attitudes and behaviours by the general factor. On the other hand, if the calculation of subscale scores are needed, then we suggest using the correlated trait model keeping in mind that the scores will not be orthogonal.

The association between the Attitude, Disclosure and Help-seeking and Social distance specific factors were weak but statistically significant that supports the notion of a shared conceptual theme. To note, the correlation coefficients in our study were lower than in Canada [19]. The results of Barney et al. demonstrated a statistically significant association between the social distance and the help-seeking inhibition; thus, people with mental health problems with higher self-stigmatising attitude are less likely to seek help for their own problems, and they also keep a higher degree of social distance [31]. Healthcare professionals and social workers, being everyday people as well, might have their own mental health and substance use problems, could experience emotional exhaustion or might have friends or family members who experience these issues [18, 32]. Moreover, healthcare providers might be aware of and affected by the stigmatising attitudes of their peers and colleagues [33].

The test-retest reliability with 1 month of a follow-up period was measured by ICC and resulted in being good (see Table 4). There have been two other studies on the test-retest reliability of the OMS-HC that reported near satisfactory reliability on the original 20-item measure of the scale [ICC was 0.66 (95% CI 0.54 to 0.75)] in Canada [17], and excellent stability was demonstrated by the Pearson’s correlation between the two measures (follow-up time of 1 week) in Italy [21].

Our findings are in correspondence with the Chilean study [22] regarding the good correlations observed between the OMS-HC and MICA-4 scale, which is a well-known measurement of the stigmatising attitude of healthcare providers. These results support that the OMS-HC is an appropriate measurement of the stigmatising attitude towards people with mental health problems.

The presented data on the psychometric properties of the OMS-HC could be integrated with the results of currently ongoing research on the stigmatisation of people with mental health problems in different occupational groups. Similarly, the OMS-HC might be used to draw conclusions about the correlations between the self-stigmatisation of people with mental illness and the stigmatising attitudes of mental health professionals, healthcare and social care providers. The Hungarian version of the OMS-HC will be useful in the evaluation of anti-stigma interventions in order to develop an effective national program to reduce the stigmatisation of patients with mental health problems.

### Limitations

Our study has some limitations that must be considered to contextualise the reported findings properly. Firstly, our study population consisted of solely practising child and adult psychiatrists; therefore, our sample does not represent diverse types of health care providers. Secondly, we aimed to contact all of the practitioners; however, some of them were not available; therefore, convenience sampling is a potential limitation of this study. Lastly, we tested the convergent validity by correlating the OMS-HC scores with the scores of only one similar questionnaire, the MICA-4, since there have not been any other available scales measuring the stigmatising attitude of healthcare professionals in Hungary. This is a limitation; it would have been beneficial to use more than one valid instrument for this purpose.

## Conclusions

In conclusion, we thoroughly examined the structure of the Hungarian version of OMS-HC among psychiatric practitioners in Hungary. Although we eliminated one item due to its poor factor loading, our results indicate that the bifactor solution explains the structure of the 15-item version of the OMS-HC appropriately; however, its model-based reliability is lower than estimated. We investigated the test-retest reliability and performed concurrent validity measures. Based on the gathered information, we concluded that the 15-item version of OMS-HC is an adequate measurement of the stigmatising attitude.

Further research with diverse samples using bifactor and model-based reliability analysis on the OMS-HC would help to determine the degree to which our findings are idiosyncratic versus universally applicable to the scale. Our findings might have important research implications in the future investigations of the construct.

## Supplementary information

**Additional file 1: Supplementary file 1.** The 15-item version of the Opening Minds Stigma Scale for Health Care Providers in English language.

**Additional file 2: Supplementary file 2.** The Hungarian translation of the 15-item version of the Opening Minds Stigma Scale for Health Care Providers.

## Data Availability

The datasets used and/or analysed during the current study are available from the corresponding author on reasonable request.

## Abbreviations

CFA: Confirmatory factor analysis
CFI: Comparative fit index
df: The degree of freedom
ECV: Explained common variance
EFA: Exploratory factor analysis
ESEM: Exploratory structural equation modelling
ICC: Intraclass correlation coefficient
KMO: Kaiser-Meyer-Olkin measure of sampling adequacy
MICA-4: The Mental Illness: Clinician’s Attitudes-4
MLMV: The maximum likelihood estimation with robust standard errors and a mean- and variance adjusted
OMS-HC: Opening Minds Stigma Scale for Health Care Providers
PUC: Percent of uncontaminated correlations
RMSA: Root mean square error of approximation
SEM: Structural equation modelling
TLI: Tucker-Lewis Index
χ^2^: Chi-square
ωH: Coefficient omega hierarchical
