# Supplementary material for: Factor structure of The Opening Minds Stigma Scale for Health Care Providers and psychometric properties of its Hungarian version
Source: BMC Psychiatry. 2020 Oct 12;20:504. doi: 10.1186/s12888-020-02902-8 (PMC7552521; doi:10.1186/s12888-020-02902-8)
Supplement: Supplementary file 1 — Additional file 1: Supplementary file 1. The 15-item version of the Opening Minds Stigma Scale for Health Care Providers in English language. [file 12888_2020_2902_MOESM1_ESM.docx]

**Opening Minds Stigma Scale for Health Care Providers**

(15-item version) [[1](#_ENREF_1), [2](#_ENREF_2)]

Please read each of the following statements and rate the extent to which it describes your feelings about people with mental illness.

Please choose from the following response options.

|  | Statement (original item number) | Strongly agree | Agree | Neither agree nor disagree | Disagree | Strongly disagree |
| --- | --- | --- | --- | --- | --- | --- |
| 1 | I am more comfortable helping a person who has a physical illness than I am helping a person who has a mental illness. (1) |  |  |  |  |  |
| 2 | If a colleague with whom I work told me they had a managed mental illness, I would be as willing to work with him/her. (3) |  |  |  |  |  |
| 3 | If I were under treatment for a mental illness I would not disclose this to any of my colleagues. (4) |  |  |  |  |  |
| 4 | I would see myself as weak if I had a mental illness and could not fix it myself. (6) |  |  |  |  |  |
| 5 | I would be reluctant to seek help if I had a mental illness. (7) |  |  |  |  |  |
| 6 | Employers should hire a person with a managed mental illness if he/she is the best person for the job. (8) |  |  |  |  |  |
| 7 | I would still go to a physician if I knew that the physician had been treated for a mental illness. (9) |  |  |  |  |  |
| 8 | If I had a mental illness, I would tell my friends. (10) |  |  |  |  |  |
| 9 | Despite my professional beliefs, I have negative reactions towards people who have mental illness. (12) |  |  |  |  |  |
| 10 | There is little I can do to help people with mental illness. (13) |  |  |  |  |  |
| 11 | More than half of people with mental illness don’t try hard enough to get better. (14) |  |  |  |  |  |
| 12 | I would not want a person with a mental illness, even if it were appropriately managed, to work with children. (17) |  |  |  |  |  |
| 13 | Health care providers do not need to be advocates for people with mental illness. (18) |  |  |  |  |  |
| 14 | I would not mind if a person with a mental illness lived next door to me. (19) |  |  |  |  |  |
| 15 | I struggle to feel compassion for a person with a mental illness. (20) |  |  |  |  |  |

1. Kassam A, Papish A, Modgill G, Patten S: The development and psychometric properties of a new scale to measure mental illness related stigma by health care providers: the Opening Minds Scale for Health Care Providers (OMS-HC). BMC psychiatry 2012, 12(1):62.

2. Modgill G, Patten SB, Knaak S, Kassam A, Szeto AC: Opening minds stigma scale for health care providers (OMS-HC): examination of psychometric properties and responsiveness. BMC psychiatry 2014, 14(1):120.
