# Supplementary material for: Factor structure of The Opening Minds Stigma Scale for Health Care Providers and psychometric properties of its Hungarian version
Source: BMC Psychiatry. 2020 Oct 12;20:504. doi: 10.1186/s12888-020-02902-8 (PMC7552521; doi:10.1186/s12888-020-02902-8)
Supplement: Supplementary file 2 — Additional file 2: Supplementary file 2. The Hungarian translation of the 15-item version of the Opening Minds Stigma Scale for Health Care Providers. [file 12888_2020_2902_MOESM2_ESM.docx]

**Az Opening Minds Stigma Skála egészségügyi dolgozóknak szóló 15 tételes változata (OMS- HC) [**[**1**](#_ENREF_1)**,** [**2**](#_ENREF_2)**]**

Kérem, hogy értékelje az állításokat aszerint, hogy mennyire ért egyet azok tartalmával.

|  |
| --- |
|  |

|  | Állítás (eredeti tételszám) | Határozottan nem értek  egyet | Nem  értek  egyet | Egyet is értek meg nem is | Egyetértek | Határozottan egyet értek |
| --- | --- | --- | --- | --- | --- | --- |
| 1 | Jobban érzem magam, ha egy olyan embernek segítek, akinek fizikális panaszai vannak, mint egy mentális betegséggel élőnek (1). |  |  |  |  |  |
| 2 | Ha egy kollégám, akivel együtt dolgozom, elmondaná nekem, hogy állapota stabil és gondozás alatt áll valamilyen mentális betegség miatt, ugyanolyan hajlandósággal dolgoznék vele együtt, mint eddig. (3) |  |  |  |  |  |
| 3 | Ha mentális betegséggel kezelnének engem, nem vállalnám fel ezt egyik kollégám előtt sem. (4) |  |  |  |  |  |
| 4 | Gyengének látnám magam, ha mentális betegségem lenne és nem tudnám magam megoldani. (6) |  |  |  |  |  |
| 5 | Vonakodva kérnék segítséget, ha mentális betegségem lenne. (7) |  |  |  |  |  |
| 6 | A munkáltatóknak a mentális betegséggel élő embert kellene felvennie, ha állapota stabil, gondozás alatt áll és ő a legjobb arra a munkára. (8) |  |  |  |  |  |
| 7 | Továbbra is járnék egy orvoshoz, ha tudomásomra jutna, hogy az orvos mentális betegség miatt korábban kezelés alatt állt. (9) |  |  |  |  |  |
| 8 | Ha mentális betegségem lenne, elmondanám a barátaimnak. (10) |  |  |  |  |  |
| 9 | A szakmai meggyőződésem ellenére negatív reakcióim vannak a mentális betegséggel élő emberekre. (12) |  |  |  |  |  |
| 10 | Keveset tudok segíteni a mentális betegséggel élő embereknek. (13) |  |  |  |  |  |
| 11 | A mentális betegséggel élő emberek több mint fele nem próbál tenni elég erősen azért, hogy jobban legyen. (14) |  |  |  |  |  |
| 12 | Nem akarnám, hogy egy olyan ember foglalkozzon gyerekekkel, aki mentális betegséggel él, még akkor sem, ha gondozásban részesül és állapota stabil. (17) |  |  |  |  |  |
| 13 | Az egészségügyi szolgáltatóknak nem szükséges a mentális betegséggel élőkkel támogatónak lenniük. (18) |  |  |  |  |  |
| 14 | Nem bánnám, ha egy mentális betegséggel élő ember lenne a szomszédom. (19) |  |  |  |  |  |
| 15 | Nehezemre sajnálatot érezni mentális betegséggel élő emberek iránt. (20) |  |  |  |  |  |

**Pontozás**

A kérdőív 3 alskálából áll. A kérdőív pszichometriai jellemzőinek vizsgálata során kapott eredmények alapján a magyar változat esetében elsősorban az összpontszám figyelembevétele részesítendő előnyben az egyes alskálák pontszámaival szemben, továbbá a 11-es (eredeti számozás szerint a 14-es) tétel elhagyását javasoljuk. Ennek megfelelően az összpontszám a 14 tétel alapján 14 ponttól 70 pontig terjedhet. Az alacsonyabb pontszám kevésbé stigmatizáló attitűdöt mutat.

A fenti állítások 1-től 5-ig terjedő Likert-skálán pontozandók.

1: Határozottan nem értek egyet, 5: Határozottan egyetértek

A *-gal jelölt állítások reverz pontozásúak:

1: Határozottan egyetértek, 5: Határozottan nem értek egyet

Alskálák és hozzájuk tartozó tételek:

**Egészségügyi szolgáltatók mentális betegséggel élő emberekhez való hozzáállása**

**1 9 10 14* 15**

**Felvállalás, segítségkérés**

**3 4 5 8***

**Távolságtartás**

**2* 6* 7* 12 13**

1. Kassam A, Papish A, Modgill G, Patten S: The development and psychometric properties of a new scale to measure mental illness related stigma by health care providers: the Opening Minds Scale for Health Care Providers (OMS-HC). BMC psychiatry 2012, 12(1):62.

2. Modgill G, Patten SB, Knaak S, Kassam A, Szeto AC: Opening minds stigma scale for health care providers (OMS-HC): examination of psychometric properties and responsiveness. BMC psychiatry 2014, 14(1):120.
